# Assessment of intimate partner violence abuse ratings by recently abused and never abused women

**DOI:** 10.1186/s12905-020-01043-0

**Published:** 2020-08-17

**Authors:** Helen E. Straus, Elizabeth H. Guonjian, Errick Christian, Rebecca R. Roberts

**Affiliations:** 1 John H Stroger Jr Hospital of Cook County, Department of Emergency Medicine, Rush University Medical Center, 1950 W Polk St., 7th floor, Chicago, 60612 USA; 2 MedStar Franklin Square Medical Center, Department of Emergency Medicine, Georgetown University School of Medicine, Washington, DC, USA

**Keywords:** Intimate partner violence, Domestic violence, Abuse ratings, Survivor experience

## Abstract

**Background:**

There are a paucity of directly reported intimate partner violence survivors’ experiences, especially in women of color. This study measures recently/currently abused women’s ratings of varied abuse events compared to ratings from never abused women.

**Methods:**

Women in a single, urban, public hospital emergency department (ED) were screened for intimate partner violence using the Abuse Assessment Screen (AAS). Two groups were identified - women abused within 1 year by an intimate partner or family member and those who screened negative for abuse. Using a two-group longitudinal survey and interview format, women completed visual analog scale ratings (0–100) for each of 20 abuse events/types. For analysis, each abuse type was placed on the 0–100 scale according to its designated rating.

**Results:**

Average age of participants in the abuse group (*n* = 30) was 33. Never abused women averaged age 50 (*n* = 32). The majority of participants were African-American: abused 67% and never abused 94%. Abused women rated name-calling (*p* < 0.02) and put-downs (*p* < 0.01) as more severe than never abused women. Other non-physical and physical forms of abuse such as threats, control, burns or forced sex were perceived more similarly between groups.

**Conclusions:**

Abused women perceive verbal abuse events differently compared to never abused women.

## Background

While there have been many projects that report aspects of intimate partner violence (IPV), few share what women themselves report about the violence [1–4]. Instead, primary emphasis has been placed upon identifying IPV [5–9], risk assessments [10] and models to better understand IPV [11–13], co-morbidities such as depression or posttraumatic stress disorder (PTSD) [14, 15] and advocacy, policy or other service/intervention models that might better assist survivors of IPV [16, 17]. Data including underserved populations are more scarce [18, 19] and the reasons for this are varied [20]. Few of these directly report the women’s perspective.

Specifically, there are a paucity of data to describe how women themselves rate the severity of abuse experiences as well as how they rank one type of abuse relative to another. There are also limited data on how women who identify as never abused might rate the severity of these same types of abuse and how their responses compare and contrast with those of recently abused women. How women rate various abuse events offers another way to distinguish the severity of abuse a woman has experienced. It also might offer a better understanding of IPV itself. While many providers of services (e.g., shelter staff and police officers, for example) may have come to some of this information anecdotally, others, such as researchers, agencies, policy experts and even medical educators developing curricula might benefit from a closer view of the experiences of these abused women. Abused women already experience “not being seen” by more influential groups [4, 21]. Underserved women likely experience this effect more distinctively [22].

For this study, we asked abused and never abused women to rate various abuse events on a 100-point scale. As they were rating each event type on a single scale, they also rated the items relative to each other.

## Methods

This pilot project took place during the second half of the three-month follow-up phase of a longitudinal intimate partner violence cohort study. The abuse type rating scale utilized in this study was introduced consecutively to the remaining cohort. The longitudinal study cohort was recruited from an urban public hospital emergency department (ED) in Chicago Illinois, with a yearly census of 120,000 visits per year. During the three-month follow-up phase, a consecutive sample of participants was asked to rate various types of violence on a 100-point scale.

Women in the longitudinal study were initially approached during a systematic sampling of days of the week and times of the day (weekday, weekend, days, evenings, and nights sampled proportionate to patient ED census patterns). Women were eligible to participate if they spoke English, were 18 years or older, were not a detainee and were not too ill (e.g.; unconscious, in severe pain or otherwise not able to participate in an informed consent process). Women who verbally consented to screening for intimate partner violence answered the 4-question Abuse Assessment Screen (AAS) developed by McFarlane et al. [23, 24]:

Have you ever been emotionally or physically abused by your partner or someone important to you?

Within the last year, have you been hit, slapped, kicked or otherwise physically hurt by someone?

Within the past year, has anyone forced you to have sexual activities?

Are you afraid of your partner or anyone you listed above?

A “yes” response to any question is considered a positive screen.

If a woman screened positive for abuse within the past year by a partner, former partner or family member on the AAS, and she completed a written informed consent process, she was assigned to the “abused women” group. If a woman screened negative for abuse, she was eligible for random selection (using a random number table) into the ‘never abused’ comparison group, after completing a written informed consent process. Each enrollee then participated in an index interview, detailing a range of health, social and economic factors. Participants also described their specific abuse experiences by one or more partners using a semi-structured interview format. Follow-up took place at 1 month and 3 months and the follow-up portion of the study took place by telephone or in-person. The study protocols were reviewed and approved by the supervising Institutional Review Board, including study risks and benefits, design and ethical concerns.

For this study, part way through the 3 month follow-up phase, a rating scale was introduced for a sample of both abused women and never abused women, for the remaining proportion of each group not yet interviewed at 3 months. Each woman was asked to rate each of a variety of types of abuse events on a 0–100 scale, with 0 being “no problem” and 100 being “the worst they could even imagine”. Women rated each item relative to the 100-point scale and were also rating each item relative to the other abuse types (on the same scale). Examples of types of abuse included: name-calling, put-downs, stalking, being hit, choked, burned, bones broken, forced sex, and being killed.

Self-reported demographic information and ratings were collected and entered into an excel spreadsheet. Because the dependent variable was not normally distributed, the Mann Whitney U Test procedure was used to determine statistically significant differences between the ratings reported by the two groups [25, 26]. Statistical analyses were conducted using SPSS version 24 (IBM Corp, 2017.). Anecdotal information was also reported.

## Results

Of the total 1365 women present in the ED during screening shifts for the longitudinal study, 1111 spoke English (81%) and 954 (86%) completed screening by a trained research assistant. Of the remainder, 41 (4%) were too ill, 78 (7%) refused screening and 38 (3%) left the ED before screening took place. Eighty-nine women screened positive for abuse within the prior year (9%). Of these, 67 agreed to participate in the longitudinal study and 65 responded to all sections of the questionnaire. Part way through the three-month follow-up, the abuse type rating scale was introduced consecutively to the remaining cohort - 30 recently abused women and 32 never abused women. All participants asked to rate types of abuse, completed ratings on the 100-point scale.

The two groups were different demographically. The mean age of women reporting recent abuse was 33.4 years compared to 50.4 years for women reporting never being abused. Women’s self-reported races and ethnicities also differed: recently abused women: 67% African-American, 17% Caucasian, 10% Hispanic and 6% multiple or other races and ethnicities, and never abused women: 94% African-American, 3% Caucasian, and 3% Hispanic. Women reporting never being abused reflect the make-up of the patient population in the ED.

Both abused and never abused women universally rated most abuse types as severe. Thirteen of the 19 abuse types rated yielded median values of 90 or above in both the abused and never abused groups (all ratings presented in Table 1). Only two of the 20 abuse types revealed statistically significant differences in ratings between the two groups. Abused women rated name-calling and put-downs higher in severity than never abused women (median 60 vs 50, U = 70.62, *p* = 0.02; 68 vs 50, U = 70.80, *p* = 0.01, respectively). All other abuse events exhibited no statistically significant differences between the two groups (Table 1).

**Table 1:** Women’s ratings of perceptions of severity by abuse type (Medians and Mann Whitney U Statistics)

	Reported Never Abused (*n* = 32)	Screened as Abused (*n* = 30)	U-value**	*P*-value
	Avg Median Rating (0–100)			
Name-calling	60	50	70.63	*0.02
Put-downs	68	50	70.84	*0.01
Threats	80	72	69.04	0.82
Jealousy	70	56.5	69.07	0.34
Stalking	80	91	68.78	0.62
Control	77	65.5	68.96	0.69
Objects Thrown	89	82	70.35	0.62
Slapped	90	91	68.05	0.26
Hit	99.5	99	66.83	0.71
Bruises	98	99	67.97	0.42
Hit w/Object	98.5	100	65.9	0.21
Fractures	100	100	61.08	0.93
Burns	100	100	44.12	0.98
Choked	98	99	47.18	0.5
Loss of Consciousness	100	100	58.91	0.84
Forced Sex	100	100	63	0.29
Forced Sex w/Objects	100	100	49.58	0.79
Held Captive	99	99.5	61.69	0.87
Knife/Gun Wound	100	100	53.73	0.08
Killed	100	100	29.7	0.24

* Denotes statistical significance at *p* = 0.05

** Differences in U-values for comparison of the same medians (i.e.: 100) occur when the distributions of perceived severity varies between groups

Besides being slapped, all physically abusive acts were seen as severe by the majority of women in both groups, thus medians were near or equal to 100. The variation in test statistics provide insight into the distributions of the ratings. The smaller the U value, the more women reported point scores of 100 in both groups. The medians of ratings by abused women for non-physical abuse events – name-calling, put-downs, threats, extreme jealousy, controlling behaviors – were generally higher/worse than those by never abused women. Stalking received a higher median rating by never abused women, however.

There were three abused women who ranked “being killed” as markedly less severe than several other physical and non-physical abuse types. One woman commented as an explanation, “It don’t matter.”.

## Discussion

This study found severity ratings of a range of abuse types by both abused and never abused women were universally high (average median score of 90 or greater) for physically and sexually violent acts. Abused women, compared to never abused women, generally rated abusive acts that did not involve a direct physical assault as more severely abusive. These differences were significant for forms of verbal abuse such as name-calling and put-downs, but not significant for threats, jealousy or controlling behaviors. The severity of abuse ratings strongly reflects their rank order on measures used by researchers, such as the Conflict Tactics Scale [27].

Differences in verbal abuse severity ratings may highlight the different experiences of abused women and never abused women when these acts occur. For abused women, these verbal assaults may occur simultaneously, as a prelude to, and/or a reminder of more severe acts of abuse. For never abused women, name-calling and put-downs might be considered in isolation and, while not healthy, may not be perceived to be as harmful as never abused women imagine physical and sexual assaults to be. It could also be that the verbal abuse itself is qualitatively different for women reporting a history of recent abuse compared to those not reporting abuse.

Three abused women ranked ‘being killed’ as not as severe as many other abuse events. This is perhaps attributable to volunteered comments by several abused women signaling hopelessness or a sense that being dead would end some of the suffering they were experiencing (“It don’t matter”). This rating and response are worth understanding better as it was unclear if fatalism is a helpful or unhelpful coping mechanism when women may be trapped in a relationship. For some women, leaving a situation may be worse. Clearly, women expressing fatalism would benefit from being assessed for depression.

Results from this study suggest that there are differences in how abused women perceive and experience different forms of abuse – and these may be different from how never abused women think about these forms of violence. Clinicians would benefit from being aware of these differences. Specifically, clinicians themselves may skew heavily towards a “never abused” perspective and may be prone to underestimating the harm of non-physical assaults in their patients. Understanding the perspective of an abused patient allows the patient to be better “heard” and supported. This alone has medical benefits for the patient but may also allow better identification and implementation of interventions for conditions such as PTSD or depression. A situation the clinician may have interpreted as more minor may now be interpreted as more serious, with a more appropriate and timely clinical response. Better understanding by clinicians leads to potentially better communication between doctor and patient and perhaps also to a better discussion of management options. In short, better clinical understanding may lead to better patient outcomes.

These results may have a bearing on policy and intervention responses to intimate partner violence. Existing research has discussed factors such as prevalence of IPV in various populations, indicators for IPV, associated diagnoses such as depression, and discussions of policy or service/intervention models. Few have parsed out the abuse experience as described by the women themselves or compared ratings of types of abuse by abused women to women who identify as never abused.

Input from abused women has implications for future IPV studies designed and conducted by never abused women (and men). Understanding and incorporating the viewpoints of abused women, especially underrepresented women, into research planning and policy formation focuses priorities and guides improved resource utilization. Integrating abused women’s perceptions of their abuse experiences leads to better policy and, one hopes, to better practices.

There are several potential limitations to these results. The study setting, primarily services an urban, low income, African-American community (as seen by the random sample of never abused women). The demographic characteristics for abused women suggests the catchment area may differ. This study sampled only English-speaking women. Some women who experienced partner abuse may have chosen not to share this fact, resulting in misclassification. Differences in responses due to in-person versus telephone interview may have been introduced. While the power was low, it is noteworthy that even with greater sample sizes it is unlikely to find differences in the perception of severe abusive acts.

## Conclusions

Non-physical abuse events such as put-downs and name-calling, are perceived to be of greater severity by abused women when compared to never abused women. Physical forms of abuse, are rated more similarly by abused and never abused women, especially the more physically damaging forms of abuse such as being burned, bones fractured, knife or gun wounds, which are ranked as highly severe by both groups.

These results offer a preliminary view of possible differences in rating abuse types, in that there is a distinct difference in how abused women perceive and rate specific non-physical forms of abuse - put-downs and name-calling - when compared to never abused women. These findings offer a basis for improved clinician-patient interactions, more targeted and effective research, and guidance for policy development.

## Data Availability

The data obtained during this study are available from the corresponding author on reasonable request.

## Abbreviations

ED: Emergency Department
AAS: Abuse Assessment Screen
IPV: Intimate Partner Violence
PTSD: Post-Traumatic Stress Disorder
